# Call for an urgent rethink of the ‘health at every size’ concept

**DOI:** 10.1186/2050-2974-2-8

**Published:** 2014-03-18

**Authors:** Amanda Sainsbury, Phillipa Hay

**Affiliations:** 1 The Boden Institute of Obesity, Nutrition, Exercise & Eating Disorders, The University of Sydney, Sydney, NSW, Australia; 2 School of Medicine and Centre for Health Research, University of Western Sydney, Locked bag 1797, 2751 Penrith, NSW, Australia; 3 School of Medicine, James Cook University, Townsville, QLD, Australia

**Keywords:** Overweight, Obesity, Metabolic health, Hypothalamic control of energy homeostasis

## The argument for an urgent rethink

When I (AS) was a child, my grandmother used to say “Don’t pull an ugly face, because if the wind changes, your face will become stuck like that”. I don’t know what evidence my grandmother had for this advice, but as a neuroscientist who studies the effects of diet on the hypothalamic control of appetite and body weight, I say “Don’t eat an ugly diet or let yourself stay fat, because if the wind changes you may become stuck with permanent obesity.”

The long-term effects of excess calories and adiposity on body weight regulation appear to have been overlooked in the fat acceptance movement that has emerged in parallel with the obesity epidemic. In this commentary I outline the possibility that excess body fat and its underlying contributors lead to permanent changes in the brain pathways that control body weight, and call for urgent reconsideration of the ‘health at every size’ concept. While I certainly agree that it is possible to have healthy behaviours that provide health benefits at a wide variety of body sizes, I disagree that it is possible to be – or to stay – truly healthy at every size.

The World Health Organisation decrees that a body mass index (BMI) of 18.5 to 24.9 kg/m^2^ is generally optimum for health, at least for Caucasians between the ages of 18 and 65 [1]. However, there are many people who are healthy who have a BMI outside this range. For instance, some people self-impose long-term calorie restriction and generally have a low BMI and exhibit metabolic benefits, in keeping with animal studies which show that long-term calorie restriction prolongs life [2]. There are also people with a BMI in the overweight (≥ 25 kg/m^2^) or obese (≥ 30 kg/m^2^) range that exhibit no signs of eminent metabolic disease whatsoever, and these are not limited to people who are body builders or elite athletes [3]. Moreover, overweight or obese people who lose as little as 5-10% of their body weight exhibit significant health gains, even if their BMI remains in the overweight or obese range. Examples include improved fertility and pregnancy outcomes [4], moderate to large clinical improvements in osteoarthritic joint pain [5], and a markedly reduced likelihood of progression to type 2 diabetes mellitus [6]. Indeed, without losing any weight at all, substantial health gains can be achieved through better diet and exercise in overweight or obese people [7].

Despite the above evidence that it certainly is possible to be healthy – or to gain health benefits – at a wide variety of BMIs, there is a large body of evidence that having a BMI outside certain limits increase the risk of health problems, hence the WHO recommendations. In general, a low BMI – an indicator of malnutrition – is associated with poor health outcomes. While the exact proportion of obese people who are metabolically healthy varies depending on what criteria are used to define both obesity and health, it is smaller than the proportion of obese people who are *not* metabolically healthy [3]. As it is currently not possible to predict which people will remain metabolically healthy despite excessive weight gain, it may be dangerous to make blanket community statements that people can have health at every size. Moreover, recent research suggests that even for obese people who are metabolically healthy, it is only a question of time before a variety of issues raise their heads, contributing to significantly greater mortality from cardiovascular disease and all other causes [8]. Whether or not a person with excess weight develops metabolic diseases such as diabetes or cardiovascular disease, sooner or later the mechanical effects of excess weight and the resultant gait abnormalities, combined with systemic inflammation, are likely to take a toll. As one example, adults who are overweight have a 2.2-fold greater chance of developing knee osteoarthritis than those with a BMI under 25 kg/m^2^, and this increases to a 2.6-fold greater risk for adults with a BMI of 30 kg/m^2^ or more [9]. Moreover, every increment in BMI contributes to escalating difficulties in performing activities of everyday life, such as walking, getting out of a chair and climbing stairs [5].

From the above considerations it is clear that overweight and obesity do not bring immediate health problems, if at all. It is only after many years that the early signs of disease start to appear, and even then, health degradation is often slow. It is thus not surprising that people frequently put off doing anything about excess weight until better conditions arise (e.g. when the children start school or leave home, when a better financial position or home is attained, after retirement, etcetera). The health at every size concept implies putting off doing anything about excess weight indefinitely, instead accepting a higher BMI and focusing on healthy behaviours.

This delay in action to combat excess weight – for whatever reason – is deeply worrying. From emerging research on dietary control of energy homeostasis in animals, it is possible that we only have a limited window of opportunity in which to do something about excess weight. After this time, carrying excess weight may become ‘hard wired’ into the parts of the brain that regulate body weight, and it may be almost impossible to make any changes at all. In normal animals, exposure to an energy dense diet that is high in fat, or high in fat and sugar – similar to the default diet of modern societies – initially leads to physiological changes that would tend to counteract weight gain, as recently reviewed [10]. Such changes include a reduced drive to eat, likely due to increased circulating concentrations of leptin, which is known to act in the brain to reduce appetite and enhance energy expenditure. Similar effects to oppose ongoing weight gain are seen in humans subject to overfeeding experiments [10]. These physiological changes facilitate fat loss and thereby promote restoration of a healthy body weight, provided that one’s appetite is heeded (e.g. don’t eat when not hungry, which can sometimes necessitate eating much lighter meals than usual, or skipping some meals altogether) [10]. This physiological propensity to defend a healthy body weight is the reason why simply making healthier food choices and eating according to appetite – without external prescriptions about what, when and how much to eat – can result in weight loss in people who are overweight or mildly obese [10], particularly when combined with conscious awareness of physical hunger signals [11]. However, problems arise when signals of reduced appetite are ignored and excess caloric intake continues unabated – as is so often the case in modern societies where delicious food is available 24 hours a day.

When animals are *chronically* exposed to caloric excess via a high fat or a high fat and high sugar diet, they eventually – after several months – develop resistance to the actions of leptin in the hypothalamus [10]. This leptin resistance contributes to a concomitantly increased drive to eat as well as accelerated body fat accretion. Chronic exposure to caloric excess in rodents has also been shown to lead to similar changes in the brain to those seen in drug addiction, and these changes are thought to contribute to the compulsive drive to overeat that these animals exhibit [12]. In sum, instead of the body fighting to oppose ongoing fat gain, as is the case during the initial stages of caloric excess and weight gain, ongoing caloric excess and fat accretion are associated with physiological changes that enable the body to pack away excess calories with heightened efficiency.

While the effects of chronic caloric excess to break the body’s natural defences against weight gain in animals are well established, the mechanisms are not yet clear. For instance, is it the increase in energy intake, the increase in dietary fat intake, or the accumulation and maintenance of greater stocks of lipid in adipose tissue – which itself is metabolically active, releasing multiple cytokines or other factors that influence hypothalamic control of energy balance – that are to blame? More importantly, and more worryingly, it is not yet known whether these detrimental effects of long-term caloric excess can be reversed by switching to a healthier diet with reduced energy intake. On the one hand, some evidence from rodents suggests that the chronic effects of overconsumption on energy homeostasis can be reversed by switching to a healthier diet [13]. On the other hand, there is evidence to suggest that long-term overconsumption may lead to permanent defence of a higher body weight (or ‘set point’), particularly in individuals that have a high genetic propensity for weight gain [14]. Moreover, new evidence shows that chronic exposure to a high fat diet in rodents leads to epigenetic changes in genes that regulate energy homeostasis, notably leptin [15]. Epidemiological evidence supports the possibility of dietary-induced epigenetic modifications in humans [16]. Epigenetic changes are changes to the DNA structure that are not encoded by the DNA sequence itself but which nonetheless result in enduring changes in gene expression and which are transmitted to subsequent generations. These findings raise the troubling possibility that long-term overconsumption contributing to maintenance of an elevated BMI leads to genetic changes that promote obesity not only in that individual, but also in any future generations.

It is currently unknown whether the effects of long-term overconsumption to promote a seemingly permanent state of obesity in rodents also occur in humans. Sadly, circumstantial evidence suggests that this may have already happened in some individuals. Making healthier food choices and eating according to appetite are examples of moderately energy restricted weight management strategies that frequently fail people who have a BMI in the very obese range. Such people frequently progress to more severe methods of weight control, such as very low calorie diets (VLCDs), which appear to instil long-lasting increases in appetite [17], a sign of the body defending and striving to restore the higher body weight from before the diet. Many people who lose weight with VLCDs or other dietary means rely on repeated dieting efforts and the long-term use of prescription anti-obesity medications in an effort to control appetite and keep weight off. These strategies also frequently fail people who are morbidly obese, and increasing numbers of people ultimately progress to bariatric surgery, the most effective – albeit still imperfect – treatment for morbid obesity.

It is distressing to hypothesise that brain changes resulting in permanent obesity may have made their way into the lives of some of those amongst us. As can be seen from the dearth of direct human data above, there are certainly gaps in the evidence for this hypothesis. However, in light of emerging evidence from animals, which show many similarities with human hypothalamic pathways controlling energy homeostasis, by the time we have robust human evidence for this hypothesis there could be great numbers of people – and possibly also their offspring – who have become hard wired for a permanent state of obesity. It is for this reason that I vehemently reject the fat acceptance movement and notions that people can have ‘health at every size’. Instead, I advocate that people who are carrying excess weight would be well served by getting help to rid themselves of it as soon as possible, while their body is still likely to be amenable to such change. As outlined above, weight gain initially activates compensatory responses – such as reduced appetite and increased energy expenditure – that actually facilitate weight loss. Nobody knows yet how long these compensatory responses (and the ‘window of opportunity for weight loss’) remain open in humans; whether it is 5 years, 30 years, or indefinitely. Moreover, nobody knows yet how this time frame differs from one person to the next. As such, the sooner any small excess in body weight is addressed, the more likely it is that it can be reversed, thereby helping to prevent the progression to a much higher BMI and morbid obesity. The danger is that if people put off losing those excess 5, 10 or 30 kilos until when ‘the time is right’, or when their doctor calls for urgent weight loss to manage a life-threatening health condition, or when they need knee replacement or any other kind of surgery, the ‘wind may have changed’, and it may be impossible to lose weight without bariatric surgery or other extreme measures that leave them feeling permanently hungry. I am in favour of healthy *behaviours* at every size, as long as they come with the definite motive of nipping excess weight in the bud – while it is still possible.

### In response

Sainsbury has presented a persuasive argument for the rethinking of the ‘health at every size’ concept. Reflecting on this it is important to consider why people have advocated for the concept. This has largely come from the fields of body image and eating disorders where the detrimental effects of over-concern about body weight are well recognised. In addition, the health effects of overweight have a strong advocacy but are often perceived as overstated.

Societal attitudes towards body weight within the normal range are also well out of step with actual health effects such that weight and/or shape overconcern has become normative amongst people of all sizes in our society (albeit at lower than levels seen in people with an eating disorder) [18]. However, mean general population body weight is increasing, leading to a ‘Catch 22’ that is likely contributing to the rise in both eating disorder related mental illness and body dissatisfaction [19]. Eating disorders and body dissatisfaction in turn are associated with impaired health related quality of life, which at the individual level may be at least as severe as impairment due to overweight or obesity [20].

We urgently need more effective ways of assisting people to manage their weight and prevent weight gain without adding to the health and social adversity people who are overweight already suffer. As Sainsbury has written here, there are likely metabolic effects of long term obesity that help explain the high failure rate of the commonly recommended ‘diet and exercise’ behavioural weight loss regimes. Early intervention and evaluation of new approaches such as that of Sainsbury [21] where there is a shift in focus from external control (i.e., eating according to prescribed portion size) to internal control (i.e., eating in response to internal hunger cues) is imperative.

However, remembering the first principal of clinical medicine ‘*first to do no harm*’, trials must ensure that weight management regimes can be used safely and not worsen or increase other problems, particularly eating disorders [22]. If one’s satiety signals no longer operate effectively it is impossible to follow internal signals. Shaming people into trying to lose weight, when the most likely outcome in the longer term may well be gaining weight, will only create or add to people’s poor self-esteem and despair and worsen physical health.

## Competing interests

AS is the author of *The Don*’*t Go Hungry Diet* (Bantam, Australia and New Zealand, 2007) and *Don*’*t Go Hungry for Life* (Bantam, Australia and New Zealand, 2011). PH is not an advocate for or representative of the “health at any/every size” movement. She is an Editor-in-Chief of the *Journal of Eating Disorders* but her views in this paper are personal and do not reflect *Journal* policy. The authors declare that they have no competing interests.

## Authors’ contributions

Both authors conceived the idea. AS wrote “The argument for” and PH wrote the response. Both authors edited the manuscript, made important revisions, and read and approved the final manuscript.
