# Correction: Call for an urgent rethink of the 'health at every size’ concept

**DOI:** 10.1186/2050-2974-2-13

**Published:** 2014-05-30

**Authors:** Amanda Sainsbury, Phillipa Hay

**Affiliations:** 1 The Boden Institute of Obesity, Nutrition, Exercise & Eating Disorders, The University of Sydney, Sydney, NSW, Australia; 2 School of Medicine and Centre for Health Research, University of Western Sydney, Locked bag 1797, 2751 Penrith, NSW, Australia; 3 School of Medicine, James Cook University, Townsville, QLD, Australia

## 

Sainsbury and Hay [1] did not cite a source for 'Health at Every Size’ in the original article. The following was sourced from the Association for Size Diversity and Health (ASDH) website, accessed on 23rd March 2014:

https://www.sizediversityandhealth.org/content.asp?id=152.

## “The Health At Every Size^®^ Principles are

1. **Weight Inclusivity:** Accept and respect the inherent diversity of body shapes and sizes and reject the idealizing or pathologizing of specific weights.

2. **Health Enhancement:** Support health policies that improve and equalize access to information and services, and personal practices that improve human well-being, including attention to individual physical, economic, social, spiritual, emotional, and other needs.

3. **Respectful Care**: Acknowledge our biases, and work to end weight discrimination, weight stigma, and weight bias. Provide information and services from an understanding that socio-economic status, race, gender, sexual orientation, age, and other identities impact weight stigma, and support environments that address these inequities.

4. **Eating for Well-being:** Promote flexible, individualized eating based on hunger, satiety, nutritional needs, and pleasure, rather than any externally regulated eating plan focused on weight control.

5. **Life-Enhancing Movement:** Support physical activities that allow people of all sizes, abilities, and interests to engage in enjoyable movement, to the degree that they choose.”

While we subscribe to and support most of the above-mentioned principles, in particular the high importance of ending weight stigma and weight bias, the article was written to address issues related to Principles 1 and 4.

With regards to Principle 1, and for the reasons outlined in our commentary, we respectfully disagree that it is possible to be - or to stay - truly healthy with body weights outside of certain thresholds.

With respect to Principle 4, we believe that externally regulated eating plans and/or an explicit focus on weight control are necessary in some situations for people to attain or maintain a healthy body weight. While we ourselves frequently draw on principles such as eating according to appetite and pleasure in our clinical practice and research, there are situations where intuitive eating plans *per se* do not result in loss of excess weight. For instance, such intuitive eating plans - while promoting psychological health - have shown disappointing results with respect to weight loss in clinical trials [2]. We believe it is most likely that intuitive eating plans need to be combined with some elements of structured, externally regulated dietary programs in order to produce reliable weight loss. Such elements may include a specific focus on choosing certain types of foods in preference to others [3,4], keeping a written record of hunger and satiety levels and eating only within externally-prescribed hunger levels [5], or heeding biofeedback on markers of physical hunger, such as blood glucose levels [6]. Given the challenges of adhering to such requirements, particularly in today’s obesogenic environment, many adults (but not children) may benefit from an explicit focus on weight loss in order to promote adherence.

In addition to a lack of robust or reliable weight loss unless combined with aspects of externally regulated eating, *ad libitum* eating plans are not suitable for people who prefer to follow more structured weight loss plans, or for whom internal hunger regulation may be disrupted, perhaps due to the hypothesised hypothalamic changes outlined in our commentary. Many people with a body mass index in the obese range may indeed benefit from severe and highly externally regulated weight loss strategies such as very low energy diets [7,8] and bariatric surgery [9,10].
